# Get secure soon: attachment in abused adolescents and young adults before and after trauma-focused cognitive processing therapy

**DOI:** 10.1007/s00787-020-01637-x

**Published:** 2020-09-12

**Authors:** Eline Rimane, Regina Steil, Babette Renneberg, Rita Rosner

**Affiliations:** 1 Department of Psychology, Catholic University Eichstätt-Ingolstadt, Ostenstr. 25, 85072 Eichstätt, Germany; 2 Department of Clinical Psychology and Psychotherapy, Institute of Psychology, Goethe University Frankfurt, Varrentrappstr. 40-42, 60486 Frankfurt am Main, Germany; 3 Department of Clinical Psychology and Psychotherapy, Freie Universitaet of Berlin, Habelschwerdter Allee 45, 14195 Berlin, Germany

**Keywords:** Attachment, Posttraumatic stress disorder, Cognitive processing therapy, Adolescents, Young adults

## Abstract

Severe posttraumatic stress symptoms (PTSS) are connected to a variety of health-related and interpersonal problems, among them are the insecure attachment orientations. However, psychotherapy seems to improve not only PTSS but also attachment insecurities. In a large multicenter, randomized clinical trial, the attachment characteristics and PTSS of 85 adolescents and young adults (aged 14–21 years) with clinically relevant abuse-related PTSS were assessed at study entry, at the end of treatment, and 3 months after the end of treatment. Participants were randomized either to a developmentally adapted cognitive processing therapy (D-CPT) or to a wait-list with treatment advice (WL/TA). The purpose of the study was to analyze the association between PTSS and attachment at study entry as well as changes in attachment during the trial. We found that attachment-related avoidance (AR avoidance) was positively associated with PTSS from both self-reports and clinician ratings, whereas attachment-related anxiety (AR anxiety) was only related to self-reported PTSS (Pearson correlation coefficients between 0.37 and 0.46). Changes in AR anxiety occurred in both conditions at some point during the study (baseline to 3-month follow-up effect size was *d* = 0.60 for D-CPT and *d* = 0.44 for WL/TA) whereas for AR avoidance, only participants in D-CPT improved significantly (baseline to 3-month follow-up effect size was *d* = 0.75). The results indicate that PTSS and attachment are connected. Positive changes in attachment insecurities brought about by trauma-focused psychotherapy seem possible.

***Trial registration***: German Clinical Trials Register (DRKS); Germanctr.de; identifier: DRKS00004787; date of registration: 18 March 2013.

**Electronic supplementary material:**

The online version of this article (10.1007/s00787-020-01637-x) contains supplementary material, which is available to authorized users.

## Introduction

Sexual and physical abuse in childhood and adolescence is associated with severe negative consequences for mental health [1, 2]. Besides an increased risk of substance abuse, suicidal behavior, affective, anxiety and personality disorders [3, 4], posttraumatic stress disorder (PTSD) is a frequently reported consequence with prevalence rates in adolescents ranging from 31% for physical abuse to 41% for rape [5]. Furthermore, child maltreatment impacts interpersonal functioning and is related to insecure attachment in children [6], adolescents [7], and adults [8]. Bowlby [9] already claimed that stressful events can negatively influence attachment status. And indeed, prospective studies confirmed an impact of stressful life experiences on attachment security (e.g. [10, 11]).

### Attachment, trauma, and posttraumatic stress symptoms

Traditionally, measuring attachment was conceptualized using a categorical approach with a focus on caregiver relationships, and assessed in interviews, primarily the Adult Attachment Interview (AAI; [12]). More recently, a focus on adult romantic relationships has evolved that uses two dimensions to describe the quality of attachment relationships: attachment-related anxiety (AR anxiety) and attachment-related avoidance (AR avoidance; [13, 14]). The anxiety dimension is associated with anxiety and vigilance concerning rejection and abandonment, whereas the avoidance dimension refers to discomfort with closeness or a reluctance to become intimate with others [14].

One of the key aspects of attachment theory of relevance for psychopathology is the hypothesis that attachment style influences an individual’s available emotion regulation strategies. During periods of stress, an anxiously attached person sees himself as relatively helpless and insistently attempts to obtain care and support from others through exaggerating threats, applying so called hyperactivating strategies. An avoidantly attached person who has no trust in others uses deactivating strategies, denying attachment needs and trying to cope with stressors on their own. Recent research showed that both of these strategies are related to psychopathology [15]. This is particularly relevant when coping with traumatic experiences. According to Mikulincer et al. [16], attachment insecurity may contribute to symptom development with hyperactivating strategies encouraging reactivation of events and intrusions and deactivating strategies encouraging avoidance of confrontation with trauma reminders. Some authors also see a protective function of AR avoidance regarding adaptation to negative life events as these strategies are an effective defense against negative thoughts and emotions [17, 18]. A recent review [19] and a meta-analysis [20], including both longitudinal and cross-sectional studies, summarized that secure attachment is related to lower levels of posttraumatic stress symptoms (PTSS) and insecure attachment to higher levels. However, studies that focus on adolescents’ and young adults’ attachment and its association with PTSS are sparse and show mixed results. Using a categorical approach, some studies reported a relationship between attachment status and PTSS in the expected direction [21–23], whereas another failed to prove this association [24]. To our knowledge, there is only one paper by Lim et al. [25] that analyzed attachment dimensions and their association with PTSS in young people. In this study both higher AR avoidance and AR anxiety were associated with more PTSS in a sample of college undergraduates who had reported at least one traumatic event in a questionnaire (mean age of 19.64 years, SD = 3.09).

No study applied an interview-based approach for the assessment of PTSS. That is worth noting as there seem to be discrepancies in the relationship between attachment and PTSS depending on the assessment modality [19, 20]: Woodward et al. [26] found a significant association between higher AR anxiety and higher self-reported PTSS, but not clinician-rated PTSS in a sample of women with experiences of intimate partner violence.

### Changes in attachment as the treatment outcome

The association between attachment, emotion regulation and PTSS further raises the question whether attachment, like the latter two, improves with effective psychotherapy. Although it is often assumed that attachment styles are stable throughout an individual’s lifespan, Bowlby [27] believed that psychotherapy could change a person’s attachment status. In a recent review, Taylor et al. [28] included 14 studies on changes in adult attachment representations after psychological treatment. The majority showed an improvement in attachment following treatment, especially an increase in secure attachment patterns and a decrease in AR anxiety. While effect sizes ranged from small to large, the findings were consistent across different assessment methods, patient groups, therapeutic interventions, and settings. However, the results on AR avoidance were less clear. Specifically for PTSD there is some evidence that trauma-focused psychotherapy can improve attachment status, but studies with a control group design are rare. Stovall-McClough and Cloitre [29] reported a positive change in attachment style measured with the AAI in female childhood abuse survivors with a diagnosis of PTSD after 16 weeks of either prolonged exposure or skills training. After treatment, participants in the exposure condition had significantly lower scores for unresolved attachment, a style associated with attachment-related trauma, than those undergoing the skills training. Muller and Rosenkranz [30] evaluated the effect of an 8-week inpatient treatment program for adults with PTSD. They also found an increase in self-reported attachment security immediately and 6 months after treatment in comparison to a wait-list group. Madigan et al. [31] analyzed the effect of trauma-focused cognitive behavior therapy (TF-CBT) on attachment status and other outcome variables in a sample of 43 pregnant adolescents with either a diagnosis of PTSD or an AAI classification of an unresolved state of mind with regard to loss or trauma. Compared to treatment as usual, they did not find an effect of TF-CBT on classifications of unresolved status at 6- and 12-months follow-up assessments.

### Present study

While results are mixed in terms of attachment, the efficacy of psychotherapy on PTSS is well established for different approaches, among them cognitive processing therapy (CPT; [32, 33]). CPT is one of the first-line choices for the treatment of PTSD for adults. It focuses on modifying the dysfunctional beliefs related to the trauma. Overall, meta-analyses show large effect sizes for cognitive interventions [34, 35], yet, they are rarely tested in adolescents [36].

In a recent multicenter randomized clinical trial, we compared a developmentally adapted CPT (D-CPT) to a wait-list condition with treatment advice (WL/TA) in a sample of adolescents and young adults (aged 14–21 years) with PTSD after abuse [37]. Compared to WL/TA, participants receiving D-CPT showed greater improvement in PTSD severity and comorbidity at posttreatment and at the 3-month follow-up. Several exploratory outcomes were recorded in the study, among them attachment and how it changes during therapy, the focus of the present article. Consequently, our first aim is to examine attachment characteristics at baseline and analyze the association between attachment and PTSS obtained from self-ratings and clinical interviews. Second, we aim to examine attachment changes during treatment, comparing attachment scores of participants in the D-CPT and WL/TA group at three time points (baseline, posttreatment and 3-month follow-up).

## Methods

### Procedure and participants

Data were collected during a multicenter randomized clinical trial. A detailed description of the trial design and the results on treatment effects regarding primary (PTSS) and secondary outcomes can be found elsewhere [37, 38].

The study was approved by the ethics committees of the universities of Eichstätt-Ingolstadt, Berlin and Frankfurt, and was therefore performed in accordance with the ethical standards laid down in the 1964 Declaration of Helsinki and its later amendments. We enrolled adolescents and young adults aged 14–21 years from July 2013 through June 2015 and obtained informed consents from all participants and, in the case of minors, from their respective parent or guardian. For inclusion, child sexual and/or physical abuse-related PTSD as a primary diagnosis was required, with a lowered threshold for avoidance symptoms (only two instead of three as defined in the DSM-IV-TR; [39]). Further inclusion criteria were sufficient German language skills, stable living conditions (i.e., no ongoing abuse and not homeless), and no or stable psychopharmacological medication (for ≥ 3 weeks). Participants were excluded in the case of current severe suicidality or severe and life-threatening suicidality or self-harming behavior within the previous 6 months. Moreover, an IQ of 75 or less, any documented pervasive developmental disorders and concurrent psychotherapy as well as a diagnosis of lifetime psychotic or bipolar disorder, current substance dependence (abstinence < 6 months), or a substance-induced disorder according to DSM-IV-TR led to exclusion.

After baseline assessments, participants were randomized either to the D-CPT or WL/TA condition. Participants in D-CPT underwent an adapted form of CPT in thirty 50-min sessions and six optional joint sessions with the caregiver or for crisis intervention. The treatment duration was 16–20 weeks (for more information on D-CPT see [40]). In the WL/TA group, adolescents received instructions for finding a psychotherapist outside the trial and were offered D-CPT after the 3-month follow-up. Approximately half (55%) of the WL/TA-participants received no further treatment, 12 reported having had psychosocial support and/or psychological or psychiatric treatment (28%), with 8 participants reporting that the trauma was addressed during treatment.

### Instruments

Attachment was assessed using the German version of the Experiences in Close Relationships—Revised Questionnaire (ECR-R; [41, 42]). The ECR-R is a widely used self-report questionnaire that assesses two dimensions of attachment in current romantic relationships: AR anxiety and AR avoidance. It comprises 36 Items (18 items for each dimension) that are rated on a 7-point Likert scale ranging from 1 (strongly disagree) to 7 (strongly agree).

For the German version of the ECR-R, good psychometrical properties regarding internal consistency (with Cronbach’s alpha of 0.92 for AR avoidance and 0.91 for AR anxiety) and construct validity were reported [42]. In the present study, Cronbach’s alpha was 0.90 for the AR anxiety and 0.93 for the AR avoidance scale. According to a recent systematic review [43], questionnaires such as the ECR are appropriate for measuring attachment in adolescents. Since there are no representative studies on adolescents, the sample of the German validation study was used to classify our results and make an apparent comparison. Ehrenthal et al. [42] analyzed results on the ECR-R of a clinical (*N* = 225) and an age- and gender-matched non-clinical (*N* = 250) sample with the clinical group showing higher scores for AR anxiety (3.71 [SD = 1.41] vs. 2.61 [SD = 1.15]) and AR avoidance (3.08 [SD = 1.27] vs. 2.46 [SD = 1.10]).

Clinician-rated PTSS were measured using a structured clinical interview, the Clinician-Administered PTSD Scale for Children and Adolescents for DSM-IV (CAPS-CA; [44]; in German, [45]). The frequency and intensity of PTSS are rated on a scale ranging from 0 (never/no problem) to 4 (most of the time/extreme), with a total score ranging from 0 to 136. In our analyses, all subscales had acceptable to good reliability (hyperarousal, *α* = 0.71; avoidance, *α* = 0.80; intrusion *α* = 0.86), with a Cronbach’s alpha of 0.90 for the overall total score.

To measure self-reported PTSS, we used the German translation [46] of the University of California at Los Angeles Post-traumatic Stress Disorder Reaction Index (UCLA-PTSD-RI; [47]; range 0–68) to assess self-reported PTSS. It uses a 5-point Likert scale ranging from 0 (none) to 4 (most of the time) for symptom frequency. Internal consistencies for the UCLA-PTSD-RI in the present sample were acceptable to good for the total score (*α* = 0.87), the avoidance (*α* = 0.81) and the intrusion subscale (*α* = 0.79). However, the hyperarousal scale had a low reliability (*α* = 0.66).

### Statistical analyses

We employed a missing completely at random (MCAR) test to examine patterns of missing values [48]. To analyze associations between PTSS and attachment at baseline, Pearson correlations were used. Statistical significance was set at the 5 percent level and thresholds were adjusted for type I errors using a sequentially rejective Bonferroni test [49].

For a first descriptive overview, changes in attachment after treatment were analyzed separately for both conditions by dependent *t*-tests and significance levels were adjusted for multiple testing as described above. Respective effect sizes were calculated using Cohen’s *d* (earlier vs. later measurements). Due to dropouts during the course of the trial and significant variation in attachment scores at baseline, we decided to analyze the effect of the different treatment conditions using multilevel modelling, which is increasingly recommended in psychotherapeutic research [50]. One major advantage over a classical ANOVA is the flexibility regarding missing data as every subject who has been observed at least once can be included in the estimation of a multilevel model for change [51, 52]. Moreover, it is possible to account for differences at baseline with a random intercept term. The assumption of random slopes allows accounting for deviations in growth rates. We chose an approach with both random intercepts and random slopes, and built two separate growth models for AR avoidance and AR anxiety with repeated measures specified at level 1 (baseline, posttreatment, follow-up) and random variation in the individual intercepts and growth rates at level 2. An unstructured covariance matrix of random effects for the intercept and slope was specified. We entered group (0 = WL/TA, 1 = D-CPT) as a covariate, and created a cross-level interaction with time to examine whether attachment changes during our treatment program. Time was inserted as distance in weeks from randomization to the respective measurement point. To test whether the growth rate changes over time we entered a quadratic term and evaluated the model fit by inspecting the Akaike’s information criterion (AIC) and Schwarz’s Bayesian information criterion (BIC) with smaller values indicating better fit.

To compare improvements in both groups, we followed the suggestion by Morris [53] and calculated an effect size based on the difference between the mean pre-post change in the treatment and control group, divided by the pooled pretest standard deviation.

All analyses were performed using IBM SPSS Statistics for Windows, Version 25.0.

## Results

For the present analyses, we only used data from trial participants for whom ECR-R scores were available at least at baseline (*N* = 85). Table 1 contains demographic data and baseline scores for attachment and PTSS. There were no significant differences between the D-CPT and WL/TA group except for higher WL/TA scores for AR anxiety (WL/TA: *M* = 4.09, SD = 1.24; D-CPT: *M* = 3.54, SD = 1.20; *t*_83_ = 2.06, *p* = 0.043) and prevalence of nicotine dependence (WL/TA: 20 [48%]; D-CPT: 11 [26%]; *χ*^2^_1_ = 4.45, *p* = 0.035).

**Table 1:** Demographic variables and baseline scores on posttraumatic stress symptoms and attachment

	Total sample (*N* = 85)	D-CPT (*n* = 43)	WL/TA (*n* = 42)	*p* value
Age, *M* (SD)	18.14 (2.23)	18.27 (2.26)	18.02 (2.22)	0.608^a^
Female, No. (%)	72 (85)	38 (88)	34 (81)	0.342^b^
Immigration background, No. (%)	21 (25)	12 (28)	9 (21)	0.489^b^
Attachment score^c^				
AR anxiety, *M* (SD)	3.81 (1.24)	3.54 (1.20)	4.09 (1.24)	0.043^a^
AR avoidance, *M* (SD)	3.71 (1.38)	3.85 (1.47)	3.56 (1.28)	0.345^a^
Posttraumatic stress symptom score				
CAPS-CA, *M* (SD)	65.46 (21.77)	65.65 (23.82)	65.26 (19.73)	0.935^a^
UCLA-PTSD-RI, *M* (SD)	42.06 (12.23)	41.16 (11.16)	42.98 (13.32)	0.498^a^
Comorbid DSM-IV disorders, *M* (SD)^c^	1.93 (1.53)	1.58 (1.20)	2.29 (1.74)	0.078^d^
Most frequent DSM-IV disorders, No. (%)				
Mood disorders	44 (52)	21 (49)	23 (55)	0.585^b^
Anxiety disorders	34 (40)	13 (30)	21 (50)	0.063^b^
Nicotine dependence	31 (36)	11 (26)	20 (48)	0.035^b^
Borderline personality disorder	14 (16)	5 (12)	9 (21)	0.223^b^
Out-of-home placement/institutional care, No. (%)	22 (26)	10 (23)	12 (29)	0.531^b^
Trauma type				
Only sexual abuse, No. (%)	16 (19)	7 (16)	9 (21)	0.544^b^
Only physical abuse, No. (%)	16 (19)	10 (23)	6 (14)	0.290^b^
Both, No. (%)	53 (62)	26 (60)	27 (64)	0.716^b^
Number of close friends, *M* (SD)	4.38 (5.21)	5.20 (6.67)	3.56 (3.02)	0.448^d^

*AR* attachment-related, *CAPS-CA* Clinician-Administered PTSD Scale for Children and Adolescents for DSM-IV, *D-CPT* Developmentally Adapted Cognitive Processing Therapy, *PTSD* posttraumatic stress disorder, *UCLA-PTSD-RI* University of California at Los Angeles Post-traumatic Stress Disorder Reaction Index, *WL/TA* wait-list/treatment advice

^a^*p* value from independent *t*-test

^b^*p* value from Pearson’s *χ*^2^ test

^c^Includes nicotine dependence and borderline personality disorder

^d^*p* value from Mann–Whitney *U* test (applied due to outliers)

The missing data were mostly due to study dropout or non-attendance at the respective measurement point. We lost 15 patients (35%) in D-CPT and 7 patients (17%) in WL/TA to the posttreatment assessment. Complete datasets were available for 49 participants (58%), 16 (19%) provided data at two of three assessment points and 20 (23%) contributed baseline data only. The MCAR test supported the assumption that data were missing completely at random (*χ*^2^_37_ = 39.93, *p* = 0.341).

### Relationship between attachment and posttraumatic stress symptoms at baseline

First, we looked at the correlations of age and gender with attachment to check for a confounding effect of these variables. There were no significant associations (data not shown). The results of the subsequent correlation analysis are given in Table 2. Both AR anxiety and AR avoidance were significantly associated with more avoidance symptoms in the CAPS-CA and UCLA-PTSD-RI as well as the total score of the UCLA-PTSD-RI. The effect sizes were in the medium to large range [54]. As AR anxiety and AR avoidance were intercorrelated (*r* = 0.24, 95% CI [0.01, 0.45], *p* = 0.026), we calculated partial correlations for the significant associations. After controlling for AR avoidance and adjusting significance thresholds, AR anxiety was still significantly correlated with the UCLA-PTSD-RI avoidance scale (*r* = 0.37, 95% CI [0.18, 0.53], *p* < 0.001) and the total score of the UCLA-PTSD-RI (*r* = 0.37, 95% CI [0.16, 0.54], *p* < 0.001), but no longer with the CAPS-CA avoidance scale (*r* = 0.23, 95% CI [0.04, 0.40], *p* = 0.035). The results were slightly different for AR avoidance after controlling for AR anxiety, resulting in significant partial correlations with the CAPS-CA avoidance scale (*r* = 0.41, 95% CI [0.23, 0.57], *p* < 0.001) and the UCLA-PTSD-RI avoidance scale (*r* = 0.46, 95% CI [0.27, 0.64], *p* < 0.001), but not with the total score of the UCLA-PTSD-RI (*r* = 0.23, 95% CI [0.02, 0.41], *p* = 0.033).

**Table 2:** Pearson’s correlation coefficients between attachment and posttraumatic stress symptoms in the CAPS-CA and UCLA-PTSD-RI (*N* = 85)

	Total		Intrusions		Avoidance		Hyperarousal	
	*r* (95% CI)	*p*	*r* (95% CI)	*p*	*r* (95% CI)	*p*	*r* (95% CI)	*p*
CAPS-CA								
AR anxiety	0.27 (0.09, 0.46)	0.011	0.10 (− 0.10, 0.30)	0.357	**0.31** (0.15, 0.48)	0.004	0.27 (0.08, 0.45)	0.013
AR avoidance	0.28 (0.07, 0.46)	0.009	0.10 (− 0.12, 0.30)	0.353	**0.46** (0.28, 0.60)	< 0.001	0.09 (− 0.12, 0.28)	0.424
UCLA-PTSD-RI								
AR anxiety	**0.42** (0.26, 0.56)	< 0.001	0.28 (0.09, 0.47)	0.009	**0.43** (0.27, 0.59)	< 0.001	0.29 (0.11, 0.46)	0.007
AR avoidance	**0.31** (0.08, 0.48)	0.004	0.14 (− 0.08, 0.35)	0.196	**0.51** (0.30, 0.67)	< 0.001	0.02 (− 0.19, 0.22)	0.893

*AR* attachment-related, *CAPS-CA* Clinician-Administered PTSD Scale for Children and Adolescents for DSM-IV, *UCLA-PTSD-RI* University of California at Los Angeles Post-traumatic Stress Disorder Reaction Index

Values in bold indicate significant correlations with *p* value < Bonferroni–Holm corrected alpha with 0.05/(16-rank + 1)

### Changes in attachment during the trial in both groups

**Fig. 1:**
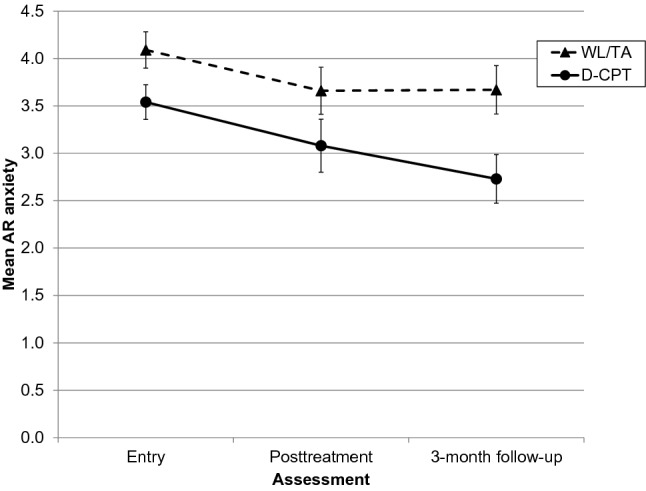
Mean scores for AR anxiety in the WL/TA and D-CPT group by assessment point. Scores range from 1 to 7 with higher scores indicating greater insecurity. Error bars represent standard errors of the mean. *AR* attachment-related, *D-CPT* developmentally adapted cognitive processing therapy, *WL/TA* wait-list/treatment advice

**Fig. 2:**
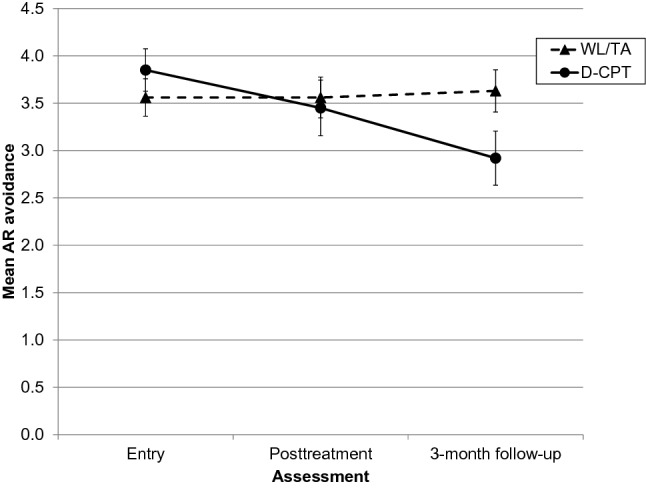
Mean scores for AR avoidance in the WL/TA and D-CPT group by assessment point. Scores range from 1 to 7 with higher scores indicating greater insecurity. Error bars represent standard errors of the mean. *AR* attachment-related, *D-CPT* developmentally adapted cognitive processing therapy, *WL/TA* wait-list/treatment advice

Posttreatment was reached on average after 27.59 (SD = 8.10) weeks in D-CPT and 22.41 (SD = 3.98) weeks in TAU. The 3-month follow-up was reached on average after 40.93 (SD = 7.63) weeks in D-CPT and 34.67 (SD = 5.72) weeks in TAU. The course of AR anxiety is presented in Fig. 1 with both groups showing a decline in the anxiety scores. For AR avoidance, only scores in the D-CPT group were reduced (see Fig. 2). For a first descriptive impression, we compared baseline, posttreatment and 3-month follow-up scores within each group. After adjusting the significance levels (alpha = 0.05/[12-rank + 1]), only the change in AR avoidance from baseline to follow-up in D-CPT remained significant (*t*_23_ = 3.66, *p* = 0.001, *d* = 0.75). However, we obtained considerable effect sizes for the WL/TA group for AR anxiety (baseline vs. posttreatment: *d* = 0.47; baseline vs. follow-up: *d* = 0.44) and for the D-CPT group for AR anxiety (posttreatment vs. follow-up: *d* = 0.42; baseline vs. follow-up: *d* = 0.60) and AR avoidance (baseline vs. posttreatment: *d* = 0.49; posttreatment vs. follow-up: *d* = 0.49). The attachment scores for the total sample and both groups at all measurements as well as results of all paired-samples *t*-tests can be found in Online Resource 1.

To obtain a better understanding of the changes in attachment, the more advanced method of multilevel modeling was used to consider all measurement points simultaneously. To test whether the growth rate changes over time we entered a quadratic term to the model and inspected the AICs and BICs. For both AR anxiety and AR avoidance the quadratic term was not significant and model fit was not improved, so it was removed from the final model.

The fixed effect parameters of the final models are presented in Table 3. The fixed or non-random part of the model includes the effects and interactions as obtained in a normal linear regression. As time was coded 0 for the first measurement and group was coded 0 for the WL/TA group, the intercepts give an estimate of the baseline scores for the WL/TA group (for AR anxiety 4.05, for AR avoidance 3.57), whereas the estimate for group indicates the approximated difference in baseline scores between WL/TA and D-CPT with − 0.51 points on the ECR-R for AR anxiety and 0.26 for AR avoidance. The estimates for the time variable approximate the change in attachment 1 week after randomization for the WL/TA group (− 0.01 points on the ECR-R for AR anxiety and − 0.00 for AR avoidance). The interaction gives an approximation of the change in the D-CPT group after 1 week of treatment, which is − 0.00 points on the AR anxiety scale and − 0.02 on the AR avoidance scale. In the final model of AR anxiety, neither group nor its interaction with time was significant. Only time significantly predicted attachment scores. For AR avoidance, the group and time variables were not significant, but there was a significant group-time interaction insofar as AR avoidance only declined in the course of D-CPT and thereafter (see also Fig. 2).

**Table 3:** Fixed effects from multilevel modelling of AR anxiety and AR avoidance

	Estimate (SE)	95% CI	*t*-value	*Df*	*p*
AR anxiety					
Intercept	4.05 (0.19)	3.68, 4.42	21.55	83.24	< 0.001
Time	− 0.01 (0.00)	− 0.02, − 0.00	− 2.30	72.18	0.024
Group	− 0.51 (0.26)	− 1.03, 0.02	− 1.92	83.39	0.058
Time × Group	− 0.00 (0.01)	− 0.02, 0.01	− 0.54	65.30	0.589
AR avoidance					
Intercept	3.57 (0.21)	3.16, 3.98	17.18	83.11	< 0.001
Time	− 0.00 (0.01)	− 0.01, 0.01	− 0.12	68.41	0.905
Group	0.26 (0.29)	− 0.32, 0.84	0.89	83.28	0.375
Time × Group	− 0.02 (0.01)	− 0.40, − 0.00	− 2.31	61.80	0.024

*AR* attachment-related

The effect sizes for improvement in AR anxiety between the two groups were *d* = 0.07 from baseline to posttreatment and *d* = 0.36 from baseline to follow-up assessments, thus indicating small effects in favor of D-CPT in the long run. For AR avoidance, the effect from baseline to posttreatment was medium (*d* = 0.65), but a large effect size was obtained from baseline to follow-up (*d* = 0.80), also in favor of D-CPT.

## Discussion

The present study evaluated attachment styles and their changes during psychotherapy in a sample of adolescents and young adults with PTSD after childhood abuse. Baseline AR anxiety and AR avoidance scores were more comparable to an adult clinical validation sample with mixed diagnoses than to a non-clinical group [42]. AR anxiety was apparently equivalent whereas AR avoidance was somewhat higher in our sample.

First, we analyzed the association between PTSS and attachment, accounting for multiple testing and intercorrelation. We found positive associations between avoidance symptoms in the UCLA-PTSD-RI and both AR anxiety and AR avoidance. Only AR avoidance was significantly related to avoidance in the CAPS-CA, and only AR anxiety was significantly related to the total score of the UCLA-PTSD-RI. Regardless of clinician rating or self-report, there was no significant relation to the intrusion or hyperarousal symptom scales. The second aim was to evaluate changes in attachment during the course of the trial and by treatment group. In both treatment conditions, participants showed a decline in AR anxiety at some point in the study. However, there was no significant effect of treatment group. For AR avoidance, only participants in D-CPT showed a significant reduction, and we found a significant group-time interaction, indicating more change in attachment avoidance in the D-CPT group.

### Results on the association between attachment and trauma symptoms differ from previous research

Some of our results are in line with recent research like the finding of a stronger association between attachment and PTSS when attachment is assessed using a self-report instrument. This is also mentioned in the meta-analysis by Woodhouse et al. [20]. There is an ongoing discussion concerning the biasing effects on the relationships between two constructs when both are measured using the same methods (e.g. [55]). According to Woodward et al. [26], self-report measures are less symptom-specific and capture more generalized distress. For PTSD, this could be specifically true. In the CAPS-CA, every symptom is rated as to whether it is functionally related to the traumatic event. In the UCLA-PTSD-RI, no such relation to trauma is assessed. For our study, this might account for the fact that the association between attachment and PTSS total score when measured using self-reports is stronger than the association between attachment and PTSS total score when measured using clinician ratings.

Most studies find a modest association between AR avoidance and PTSS and instead a stronger association between AR anxiety and PTSS [19, 20]. We found mixed results that differed by assessment method. Only the association between AR avoidance and posttraumatic avoidance symptoms was obtained with both instruments. Compared to recent research, our results do not support the assumptions of the emotion regulation model proposed by Mikulincer et al. [56] or the idea of Fraley and Bonanno [17] of avoidance having a protective function. However, directions of correlations are unclear. The rather strong association between AR avoidance and posttraumatic avoidance may imply, on the one hand, that attachment insecurities lead to an increase in posttraumatic avoidance, and, on the other, that posttraumatic avoidance leads to attachment insecurities in close relationships. The comparison of our results with existing research is difficult, as most of the studies included adults and comparable adolescents studies used different assessment methods and did not report dimensional attachment scores [21–24]. One comparable study might be the work of Lim et al. [25] that used the ECR-R in a sample of college undergraduates with various traumatic events. Compared to our sample with clinically significant PTSS, participants showed slightly lower AR anxiety (3.39 vs. 3.81) and much lower AR avoidance (2.94 vs. 3.71) scores. This is not surprising given the relationship between attachment and psychopathology. Lim et al. [25] also found a relationship between AR avoidance and AR anxiety with self-reported PTSS. However, the correlation coefficients were lower (0.29 for anxiety and 0.20 for avoidance) than in the current study, and correlations with PTSS subscales were not reported.

### Attachment-related avoidance improves during trauma-focused treatment

To evaluate the effect of D-CPT compared to WL/TA on attachment scores, we compared AR anxiety and AR avoidance of participants in both groups at baseline, posttreatment, and 3-month follow-up. In terms of AR anxiety, we did not find an effect for group on reduction of AR anxiety. For AR avoidance, we found a significant group-time interaction; only participants in D-CPT improved significantly from baseline to follow-up. Taylor et al. [28] reported consistent findings for a decrease in AR anxiety and less consistent findings for AR avoidance in their review. However, they included different psychological therapies for a range of disorders. Regarding trauma-focused treatments in particular, we found five other studies, but all except one used either the AAI [29, 31] or a projective test [57, 58]. Muller and Rosenkranz [30], who chose a dimensional approach to measure attachment (though not the ECR-R) and applied an intervention focusing mainly on skills and on the establishment of a sense of safety, found that both AR anxiety and AR avoidance decreased during therapy but only changes in AR anxiety remained stable 6 months after the end of the program. The authors conclude that in contrast to AR anxiety, AR avoidance is more resistant to enduring improvements and refer to Fraley and Shaver [14] who believe that AR anxiety is more sensitive to changes in relational experiences than attachment avoidance*.* In our study, AR anxiety improved in both groups. However, with nearly half of our WL/TA participants receiving some kind of support or treatment and all of them having several appointments for study assessments, the participants in this group experienced interpersonal contacts that may have attenuated AR anxiety. For AR avoidance, there was a clear advantage of D-CPT. Only 8 patients in the WL/TA group reported that the trauma was directly targeted during their treatment. The focus on exposure with trauma-related memories and cognitions in D-CPT could perhaps explain the larger improvement in AR avoidance in this group.

### Strengths and limitations

To our knowledge, this is the first study to evaluate the effect of a trauma-focused behavioral therapy on the two attachment dimensions, AR anxiety and AR avoidance, in a sample of adolescents with PTSD. We were able to monitor changes in attachment for individuals being treated with an effective therapy approach in comparison to a control group where most participants did not receive any evidence-based treatment or no treatment at all. We only included participants with clinically significant PTSS that was assessed using clinician ratings and self-report.

Despite these strengths, our study has several limitations. First, although our RCT has one of the biggest samples within PTSD trials on young patients, our sample size was quite small. For growth models, a sample size of at least 100 is preferred, but also models with much smaller sample sizes have been fitted successfully [59]. As our sample size was below 100, we decided to reduce the complexity of the model and did not include any additional variables such as age, sex, type of abuse (sexual vs. physical), etc., that might have been of interest. We would also like to stress that 26% of our sample lived with out-of-home placements/in institutional care, so we were faced with a sample at high risk of attachment difficulties (e.g. [60]). Furthermore, a wait-list condition might lead to larger effect sizes. In our study, however, we found a large effect size for the interview-rated PTSS in the WL/TA group (for details see [37]) with one-third of WL/TA-participants receiving some kind of professional support. Some points regarding our assessment instruments need to be discussed: First, reliability was relatively low for the hyperarousal scale of the UCLA-PTSD-RI and this means that any conclusions about hyperarousal symptoms should be drawn with caution. Furthermore, we chose a self-report approach to assess attachment for reasons of economy, comparability, psychometry and fit to the characteristics of this age group. However, it should be considered that self-reports like the ECR-R can be biased, since e.g. attachment style affects the information that people remember about their relationships [61]. In addition, there may have been method bias when comparing the results of self-report measures. Furthermore, although Jewell et al. [43] see the ECR as an appropriate instrument for the assessment of attachment in young people, they also state that this construct is difficult to measure in adolescence and necessitate the use of developmentally adapted measures. Wilkinson [62] and Brenning et al. [63] modified the ECR-R for use with adolescents but, for the time being, no validated German version is available. Thus, we decided to use the original, translated and validated measure. Finally, it has to be emphasized that the question of directionality of the identified relationship between attachment and PTSS remains unclear and further analyses including other treatment outcomes might have complementary findings.

### Clinical implications

The consideration of attachment in psychotherapy could produce beneficial effects for therapists and patients. On one hand, therapists should be aware that adolescents with PTSD after sexual and physical abuse display elevated AR anxiety and AR avoidance, and that these behavioral patterns may influence the outcome of psychotherapy. Sensitivity for attachment issues may help therapists to establish an optimum therapeutic alliance. On the other hand, attachment insecurities seem to improve with psychotherapy. The perspective of experiencing an improvement in interpersonal relationships might increase treatment motivation. However, especially in this sample of adolescents and young adults with abuse experiences, it should be noted that the aim of treatment is to change the representation of attachment and not to enter directly into a new intimate relationship. This population has an elevated risk of starting relationships with abusive partners, so a discussion about partner choice is crucial in this treatment. Nevertheless, attachment issues are highly relevant for case conceptualization, treatment planning and understanding the process of change [64].

## Conclusion

This is one of the few studies on attachment in adolescents diagnosed with PTSD. The findings shed light on the association between attachment insecurities and PTSS and its changeability during psychotherapy. Future studies should also use attachment measures based on blind expert ratings and should include significant persons in the patient’s environment in the assessments, e.g. via proxy-reported measures. Moreover, it would be interesting to also address comorbid disorders and to consider the relationship between early attachment, complex PTSD and reversibility through treatment. It could be of particular interest to identify which specific interventions are responsible for inducing changes in attachment. D-CPT combines classical CPT with techniques for enhancing treatment commitment and improving emotion management strategies while taking developmental tasks into account. Thus, it is still unclear whether CPT alone, one of these additional elements or a combination thereof encourages changes in self-reported attachment.

## Electronic supplementary material

Below is the link to the electronic supplementary material.

Supplementary file1 (PDF 44 kb)
