# Supplementary material for: Get secure soon: attachment in abused adolescents and young adults before and after trauma-focused cognitive processing therapy
Source: Eur Child Adolesc Psychiatry. 2020 Sep 12;30(10):1591–601. doi: 10.1007/s00787-020-01637-x (PMC8505313; doi:10.1007/s00787-020-01637-x)
Supplement: Supplementary file 1 — Supplementary file1 (PDF 44 kb) [file 787_2020_1637_MOESM1_ESM.pdf]

Get secure soon: attachment in abused adolescents and young adults before and after trauma-focused cognitive processing therapy

Eline Rimane, Regina Steil, Babette Renneberg, Rita Rosner

Corresponding author: Eline Rimane, Dipl.-Psych., Department of Psychology, Catholic University Eichstätt-Ingolstadt, Ostenstr. 25, 85072 Eichstätt, Germany (eline.rimane@ku.de)

Table 1 AR anxiety and AR avoidance at different assessment points

	N	Total	*n*	D-CPT	*n*	WL/TA
Baseline						
AR anxiety, *M* (SD)	85	3.81 (1.24)	43	3.54 (1.20)	42	4.09 (1.24)
AR avoidance, *M* (SD)	85	3.71 (1.38)	43	3.85 (1.47)	42	3.56 (1.28)
Posttreatment						
AR anxiety, *M* (SD)	57	3.39 (1.42)	27	3.08 (1.45)	30	3.66 (1.36)
AR avoidance, *M* (SD)	57	3.50 (1.34)	27	3.45 (1.53)	30	3.56 (1.18)
3-month follow-up						
AR anxiety, *M* (SD)	57	3.27 (1.45)	24	2.73 (1.26)	33	3.67 (1.46)
AR avoidance, *M* (SD)	57	3.33 (1.36)	24	2.92 (1.40)	33	3.63 (1.28)

Abbreviations: AR, attachment-related; D-CPT, Developmentally Adapted Cognitive Processing Therapy; WL/TA, wait-list/treatment advice.

Table 2 Within-group comparisons of baseline, posttreatment and 3-month follow-up assessments for AR anxiety and AR avoidance (using paired-samples t-tests)

	D-CPT				WL/TA			
	t	*df*	*p*	*d*	*t*	*df*	*p*	*d*
Baseline vs. posttreatment								
AR anxiety	1.51	26	.142	0.29	2.55	29	.016	0.47
AR avoidance	2.57	26	.016	0.49	-0.34	29	.736	-0.06
Posttreatment vs. follow-up								
AR anxiety	1.94	20	.067	0.42	-0.26	27	.794	-0.05
AR avoidance	2.25	20	.036	0.49	-0.06	27	.956	-0.01
Baseline vs. follow-up								
AR anxiety	2.95	23	.007	0.60	2.50	32	.018	0.44
AR avoidance	**3.66**	23	.001	0.75	0.50	32	.622	0.09

Abbreviations: AR, attachment-related; D-CPT, Developmentally Adapted Cognitive Processing Therapy; WL/TA, wait-list/treatment advice.

Values in bold indicate significant correlations with *p*-value < Bonferroni-Holm corrected alpha with 0.05/(12-rank+1).
